# Diagnostic and prognostic prediction using gene expression profiles in high-dimensional microarray data

**DOI:** 10.1038/sj.bjc.6601326

**Published:** 2003-10-28

**Authors:** R Simon

**Affiliations:** 1 Biometric Research Branch, Division of Cancer Treatment & Diagnosis, National Cancer Institute, 9000 Rockville Pike, MSC #7434, Bethesda, MD 20892, USA

**Keywords:** microarray, gene expression profiling, diagnostic classification, prognostic classification

## Abstract

DNA microarrays are a potentially powerful technology for improving diagnostic classification, treatment selection and therapeutics development. There are, however, many potential pitfalls in the use of microarrays that result in false leads and erroneous conclusions. This paper provides a review of the key features to be observed in developing diagnostic and prognostic classification systems based on gene expression profiling and some of the pitfalls to be aware of in reading reports of microarray-based studies.

The literature of prognostic markers for patients with cancer is vast, but most proposed markers are either not reproducibly established as prognostic in a medically relevant context or not widely used in clinical practice. Broadly accepted good practice standards for the development of prognostic markers and prognostic classification systems do not exist (Simon and Altman, 1994). Some of the problems that exist in the prognostic marker literature derive from the nonprospective nature of most marker studies. Clinical drug trials are generally prospective, with patient selection criteria, primary end point, hypotheses and analysis plan specified in advance in a written protocol. The consumers of clinical trial reports have been educated to be skeptical of *data dredging* to find something ‘statistically significant’ to report in clinical trials. They are skeptical of analyses with multiple end points or multiple subsets, knowing that the chances of erroneous conclusions increase rapidly once one leaves the context of a focused single hypothesis clinical trial. Prognostic marker studies are generally performed with no written protocol, no eligibility criteria, no primary end point or hypotheses and no defined analysis plan. The analysis is often much less structured, the patient population more heterogeneous, and there are many subset analyses.

Most of the problems that have hindered the development and acceptance of prognostic markers exist also for classification systems based on DNA microarray expression profiles. For example, there are multiple platforms and protocols for measuring expression profiles, and most studies do not evaluate either interlaboratory assay reproducibility or intralaboratory reproducibility on multiple samples of the same tissue specimen. Some of the problems that have hindered the development of reliable prognostic markers are exacerbated in DNA microarray studies. Owing to the number of genes available for analysis, microarray data can be a veritable fountain of false findings unless appropriate statistical methods are utilised.

DNA microarray experiments require planning (Simon and Dobbin, 2002). Planning is driven by experimental objectives. Good DNA microarray experiments have clear objectives. These objectives do not usually involve gene-specific mechanistic hypotheses. Nevertheless, successful studies are not unfocused searches for interesting patterns that provide clear answers to un-asked questions. One type of objective commonly encountered in DNA microarray experiments is identification of genes differentially expressed among predefined classes of samples. We will refer to this as *class comparison*. A related objective that is often relevant for medical studies is the development of a mathematical function that can accurately predict the biologic group, diagnostic category or prognostic stage of a patient based on an expression profile of the diseased tissue from that patient. This will be referred to as *class prediction*. With these objectives, the sample classes are defined in advance independently of the gene expression data.

The classes used for class comparison may represent different tumour types and the focus may be identifying the gene expression correlates of that tumour type classification. The classes in class prediction studies often represent prognostic or response groups for patients who have received a particular therapy. For example, Wang *et al* (2002) developed a gene-expression-based predictor of whether a patient with advanced melanoma would respond to IL2-based treatment. Such predictors can be developed using data from phase II studies and the predictors can be used to select patients for phase III trials of the new treatment.

In some problems of prognostic prediction, the outcome is a continuous measurement rather than a categorical class variable. For example, Rosenwald *et al* (2002) and Shipp *et al* (2002) developed gene-expression-based prognostic predictors for patients with diffuse large B-cell lymphoma receiving doxorubicin-based combination chemotherapy. Many of the methodologic issues pertinent to class prediction are also important for prognostic prediction. For simplicity of exposition, however, we will primarily refer to the problem of class prediction here.

*Class discovery* is fundamentally different from class comparison or class prediction in that no classes are predefined. Usually, class discovery in cancer studies is for the purpose of determining whether discrete subsets of a disease entity can be defined based on the gene expression profiles. This is different from determining whether the expression profiles correlate in some way with some already known diagnostic classification. An example of class discovery was the study by Bittner *et al* (2000) examining expression profiles for advanced melanomas. Alizadeh *et al* (2000) also performed class discovery in examining the expression profiles of patients with diffuse large B-cell lymphoma.

Cluster analysis is useful for class discovery, but is generally not appropriate or effective for class comparison or class prediction. Cluster analysis refers to an extensive set of methods of partitioning samples into groups based on the pairwise distances of their expression profiles. Cluster analysis is considered an *unsupervised* method because class membership indicators are not utilised. The pairwise distance measures between expression profiles are generally computed with regard to the complete set of genes represented on the array, or those that are well measured with sufficient intensity. The genes that distinguish particular classes may be few in number relative to the full set of genes. Consequently, the pairwise distances used in cluster analysis will often not reflect the influence of these relevant genes. This accounts for the poor results often obtained in attempting to use cluster analysis for class prediction. Even if the classes do group into clusters based on an unsupervised distance metric, the analysis does not provide a useful class predictor that can be used for new cases.

Our objective here is to highlight important aspects of the process of developing and evaluating gene-expression-based class predictors. Many of the recommended methods for developing and evaluating gene-expression-based classifiers are available in the BRB-ArrayTools software available without charge for noncommercial purposes from the National Cancer Institute (Simon and Peng-Lam, 2003).

## COMPONENTS OF CLASS PREDICTION

One component of developing a class predictor is determining which genes to include in the predictor. This is generally called ‘feature selection’ or, in the context of microarray prediction, gene selection. Usually, including too many ‘noise variables’ in the predictor reduces the accuracy of prediction. It also makes interpretation and future use of the predictor more difficult. A noise variable is a variable that is not related to the class being predicted. Feature selection is particularly important in microarray studies because the number of noise variables may be orders of magnitude greater than the number of relevant variables. The influence of the genes that actually distinguish the classes may be lost among the noise of the more numerous noise genes unless we select the informative genes to be utilised by the class predictor.

The second main component of a class predictor is complete specification of the mathematical function that will provide a predicted class label for any given expression vector. There are many kinds of predictor functions as will be discussed in section ‘Class prediction algorithm’.

The third component of developing a class predictor is parameter estimation. Most kinds of predictors have parameters that must be assigned values before the predictor is fully specified. These parameters are in many ways equivalent to the regression coefficients of ordinary linear regression. The machine learning literature calls the process of specifying the parameters ‘learning the data’ but it is equivalent to fitting the parameters of a nonlinear regression model. Even neural network models are really nonlinear regression models, although they are often represented as something more exotic (Faraggi and Simon, 1995).

For many kinds of predictors, there is also a cut-point that must be specified for translating a quantitative predictive index into a predicted class label (e.g., 0 or 1) for binary class prediction problems. Completely specifying the predictor means specifying all of these aspects of the predictor, the type of predictor, the genes included and the values of all parameters.

## ESTIMATING ACCURACY OF A CLASS PREDICTOR

The most important requirement for a class predictor is that it predict accurately. Neils Bohr supposedly said: ‘Prediction is difficult, particularly the future.’ But it is the future that we want to predict. We want to be able to predict class membership for future samples whose class membership we do not know. For example, Wang *et al* (2002) wanted to predict response to IL2-based treatment as a way of selecting treatments for individual patients in the future. The current samples are available to help us estimate how accurate the class predictor we develop will be for future predictions. We must be careful about how we use the current samples to estimate accuracy, however, or our estimates may be erroneous and misleading, as discussed below.

How can we develop a proper estimate of the accuracy of class prediction for future samples? For a future sample, we will apply a fully specified predictor developed using the data available today. If we are to emulate the future predictive setting in developing our estimate of predictive accuracy, we must set aside some of our samples and make them completely inaccessible until we have a fully specified predictor that has been developed from scratch without utilising those set aside samples.

To estimate properly the accuracy of a predictor for future samples, the current set of samples must be partitioned into a training set and a separate test set. The test set emulates the set of future samples for which class labels are to be predicted. Consequently, the test samples cannot be used in any way for the development of the prediction model. This means that the test samples cannot be used for estimating the parameters of the model and they cannot be used for selecting the gene set to be used in the model. It is this latter point that is often overlooked.

The most straightforward method of estimating the accuracy of future prediction is the *split-sample* method of partitioning the set of samples into a training set and a test set as described in the previous paragraph. Rosenwald *et al* (2002) used this approach successfully in their international study of prognostic prediction for large cell lymphoma. They used two-thirds of their samples as a training set. Multiple kinds of predictors were studied on the training set. When the collaborators of that study agreed on a fully specified prediction model, they accessed the test set for the first time. On the test set there was no adjustment of the model or fitting of parameters. They merely used the samples in the test set to evaluate the predictions of the model that was completely specified using only the training data.

**Figure 1:**
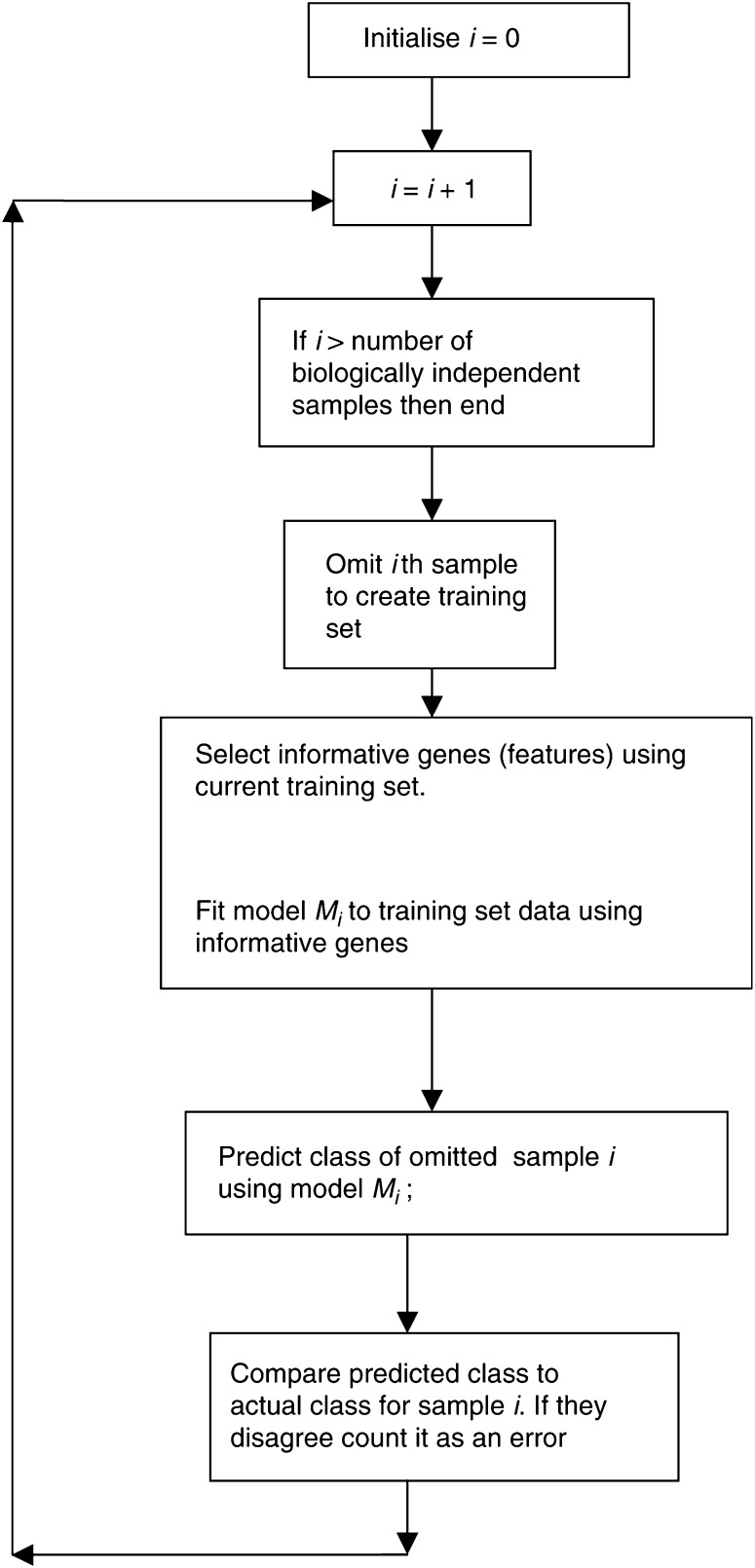
Schematic diagram of leave-one-out cross-validation (LOOCV).

*Cross-validation* is an alternative to the split sample method of estimating prediction accuracy (Radmacher *et al*, 2002). There are several forms of cross-validation. Here, we will describe *leave-one-out cross-validation (LOOCV)* as depicted in Figure 1

. LOOCV starts like split-sample cross-validation in forming a training set of samples and a test set. With LOOCV, however, the test set consists of only a single sample; the rest of the samples are placed in the training set. The sample in the test set is placed aside and not utilised at all in the development of the class prediction model. Using only the training set, the informative genes are selected and the parameters of the model are fit to the data. Let us call *M*_1_ the model developed with sample 1 in the test set. When this model is fully developed, it is used to predict the class of sample 1. This prediction is made using the expression profile of sample 1, but obviously without using knowledge of the true class of sample 1. Symbolically, if *x*_1_ denotes the complete expression profile of sample 1, then we apply model *M*_1_ to *x*_1_ to obtain a predicted class *ĉ*_1_. This predicted class is compared to the true class label *c*_1_ of sample 1. If they disagree, then the prediction is in error. Then a new training set – test set partition is created. This time sample 2 is placed in the test set and all of the other samples, including sample 1, are placed in the training set. A new model is constructed from scratch using the samples in the new training set. Call this model *M*_2_. Model *M*_2_ will generally not contain the same genes as model *M*_1_. Although the same algorithm for gene selection and parameter estimation is used, since model *M*_2_ is constructed from scratch on the new training set, it will in general not contain exactly the same gene set as *M*_1_. After creating *M*_2_, it is applied to the expression profile *x*_2_ of the sample in the new test set to obtain a predicted class *ĉ*_2_. If this predicted class does not agree with the true class label *c*_2_ of the second sample, then the prediction is in error.

The process described in the previous paragraph is repeated *n* times, where *n* is the number of biologically independent samples. Each time it is applied, a different sample is used to form the single-sample test set. During the steps, *n* different models are created and each one is used to predict the class of the omitted sample. The number of prediction errors is totalled and reported as the leave-one-out cross-validated estimate of the prediction error.

At the end of the LOOCV procedure, you have constructed *n* different models. They were only constructed in order to estimate the prediction error associated with the type of model constructed. The model that would be used for future predictions is the one constructed using all *n* samples. That is the best model for future prediction and the one that should be reported in the publication. The cross-validated error rate is an estimate of the error rate to be expected in use of this model for future samples assuming that the relationship between class and expression profile is the same for future samples as for the currently available samples. With two classes, one can use a similar approach to obtain cross-validated estimates of the sensitivity, specificity.

The cross-validated prediction error is an estimate of the prediction error associated with the algorithm for model building used. It is not an estimate constructed for a specific model. If we use all of the data to select genes and construct a specific model, there is no independent data left to estimate validly prediction error. A commonly used invalid estimate is called the *resubstitution* estimate. You use all the samples to develop a model *M*. Then you predict the class of each sample *i* using its expression profile *x*_*i*_; *ĉ*_*i*_=*M*(*x*_*i*_). The predicted class labels are compared to the true class labels and the number of errors are totaled.

Simon *et al* (2003) performed a simulation to examine the bias in estimated error rates for class prediction (see their supplemental information for a full description of the simulation). Two types of LOOCV were studied: one with removal of the left out specimen prior to selection of differentially expressed genes and one with removal of the left out specimen prior to computation of gene weights and the prediction rule but after gene selection. They also computed the *resubstitution* estimate of the error rate. In a simulated data set, 20 gene expression profiles of length 6000 were randomly generated from the same distribution. In all, 10 profiles were arbitrarily assigned to ‘Class 1’ and the other 10 to ‘Class 2’, creating an artificial separation of the profiles into two classes. Since no true underlying difference exists between the two classes, class prediction will perform no better than a random guess for future biologically independent samples. Hence, the estimated error rates for simulated data sets should be centred around 0.5 (i.e., 10 misclassifications out of 20).

Without using cross-validation, an astounding 98.2% of the simulated data sets resulted in an estimate of zero misclassifications even though no true underlying difference exists between the two groups. Moreover, the maximum number of misclassified profiles using the resubstitution method was only one. Cross-validating the prediction rule after selection of differentially expressed genes from the full data set does little to correct the bias of the resubstitution estimator: 90.2% of simulated data sets still resulted in zero misclassifications. It is not until gene selection is also subjected to cross-validation that the estimate of error rate was in line with our expectation: the median number of misclassified profiles jumps to 11, although the range is large (0–20).

The simulation results underscore the importance of cross-validating all steps of predictor construction in estimating the error rate. A study of breast cancer also illustrates the point: van't Veer *et al* (2002) predicted clinical outcome of patients with axillary node-negative breast cancer (metastatic disease within 5 years *vs* disease-free at 5 years) from gene expression profiles, first using the resubstitution method and then using a fully cross-validated approach. The investigators controlled the number of misclassified recurrent cases (i.e., the sensitivity of the test) in both situations, so here we focus attention on the difference in estimated error rates for the disease-free cases. The improperly cross-validated method and the properly cross-validation result in estimated error rates of 27% (12 out of 44) and 41% (18 out of 44), respectively. The improperly cross-validated method results in a seriously biased underestimate of the error rate, probably largely due to overfitting the predictor to the specific data set. While van't Veer *et al* (2002) report both estimates of the error rate, the properly cross-validated estimate was reported only in the supplemental results section on the website and the invalid estimate received more attention. Another example of this occurred in a study where classification trees were built from gene expression data to classify specimens as normal colon or colon cancer (Zhang *et al*, 2001). The authors used a procedure that only cross-validated steps that occurred *after* selection of genes for inclusion in the predictor from the full data set. As our simulation shows, not subjecting gene selection to cross-validation can result in a large bias. Other examples of incorrect use of cross-validation are described by Ambroise and McLachlan (2002). There are numerous articles in the most prominent journals, written by both biologists and by methodologists, which make claims for gene expression classifiers and for new classification algorithms, which are invalid because they have cross-validated improperly.

## CLASS PREDICTION ALGORITHMS

### Feature selection

The most commonly used approach to feature selection is to identify the genes that are differentially expressed among the classes when considered individually. For example, if there are two classes, one can compute a *t*-test or a Mann–Whitney test for each gene. The log-ratios or log-signals are generally used as the basis of the statistical significance tests. The genes that are significantly differentially expressed at a specified significance level are selected for inclusion in the class predictor. The stringency of the significance level used controls the number of genes that are included in the model. If one wants a class predictor based on a small number of genes, the threshold significance level is made very small. Issues of multiple testing or false positives are not really relevant, however, because the objective is just to select features for inclusion in the model; no particular claim is made about the selected genes. Similarly, it does not really matter whether the assumptions of the *t*-test are strictly satisfied, because the *P*-values are merely used as a convenient index for selecting genes. Some methods do not use *P*-values at all but merely select the *n* most differentially expressed genes, and specify *n* arbitrarily.

Several authors have developed methods to identify optimal sets of genes that together provide good discrimination of the classes (Bo and Jonassen, 2002; Kim *et al*, 2002; Deutsch, 2003; Ooi and Tan, 2003). These algorithms are generally very computationally intensive, some requiring a large cluster of parallel computers. Unfortunately, it is not clear whether the increased computational effort of these methods is warranted. In some cases, the claims made do not appear to be based on properly cross-validated calculations; all of the data being used to select the genes and cross-validation used only for fitting the parameters of the model. Studies comparing the performance of such methods to the simpler univariate methods are needed.

Some investigators have used linear combinations of gene expression values as predictors (Khan *et al*, 2001; West *et al*, 2001). *Principal components* are the orthogonal linear combinations of the genes showing the greatest variability among the cases. The principal components are sometimes referred to as singular values (Alter *et al*, 2000). Using principal components as predictive features provides a vast reduction in the dimension of the expression data, but has two serious limitations. One is that the principal components are not necessarily good predictors. The second problem is that measuring the principal components requires measuring expression of all the genes and this may not be desirable for clinical application.

### Algorithm specification

Many algorithms have been used effectively with DNA microarray data for class prediction. Dudoit *et al* (2002) compared several algorithms using publicly available data sets. The algorithms compared included several variants of linear discriminant analysis, nearest neighbour classification and several variants of classification trees. A linear discriminant is a function


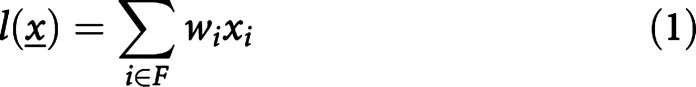


where *x*_*i*_ denotes the log-ratio or log-signal for the *i*th gene, *w*_*i*_ is the weight given to that gene, and the summation is over the set *F* of features (genes) selected for inclusion in the class predictor. For a two-class problem, there is a threshold value *d*, and a sample with expression profile defined by a vector *x* of values is predicted to be in class 1 or 2 depending on whether *l*(*x*) as computed from equation 1 is less than the threshold *d* or greater than *d* respectively.

There are a large number of class predictors based on linear discriminants of the form shown in (1). They differ with regard to how the weights are determined. The oldest form of linear discriminant is Fisher's linear discriminant. The weights are selected so that the mean value of *l*(*x*) in class 1 is maximally different from the mean value of *l*(*x*) in class 2. The squared difference in means divided by the pooled estimate of the within-class variance of *l*(*x*) was the specific measure used by Fisher. To compute these weights, one must estimate the correlation between all pairs of genes that were selected in the feature selection step. The study by Dudoit *et al* indicated that Fisher's linear discriminant analysis did not perform well unless the number of selected genes was small relative to the number of samples. The reason is that in other cases there are too many correlations to estimate and the method tends to be unstable and overfit the data.

Diagonal linear discriminant analysis is a special case of Fisher linear discriminant analysis in which the correlation among genes is ignored. By ignoring such correlations, one avoids having to estimate many parameters, and obtains a method that performs better when the number of samples is small. Golub's weighted voting method (Golub *et al*, 1999) and the Compound Covariate Predictor of Radmacher *et al* (2002) are similar to diagonal linear discriminant analysis and tend to perform very well when the number of samples is small. They compute the weights based on the univariate prediction strength of individual genes and ignore correlations among the genes.

Support vector machines (SVMs) are very popular in the machine learning literature. Although they sound very exotic, linear kernel support vector machines do class prediction using a predictor of the form of . The weights are determined by optimising a misclassification rate criterion, however, instead of a least-squares criterion as in linear discriminant analysis (Ramaswamy *et al*, 2001). Although there are more complex forms of support vector machines, they appear to be inferior to linear kernel SVMs for class prediction with large numbers of genes (Ben-Dor *et al*, 2000).

Khan *et al* (2001) reported accurate class prediction among small, round blue cell tumours of childhood using an artificial neural network (ANN). The inputs to the ANN were the first 10 principal components of the genes, that is, the 10 orthogonal linear combinations of the genes that accounted for most of the variability in gene expression among samples. Their neural network used a linear transfer function with no hidden layer and hence it was a linear classifier of the form of equation 1. Most true artificial neural networks have hidden nodes, nonlinear transfer functions and individual features as inputs. Such functions would likely not perform as well as the linear model of Khan *et al* because of the number of parameters to be estimated and the greater opportunity for overfitting the data.

In the study of Dudoit *et al* (2002), the simplest methods, diagonal linear discriminant analysis, and nearest neighbour classification performed as well or better than the more complex methods. Nearest neighbour classification is defined as follows. It depends on a feature set *F* of genes selected to be useful for discriminating the classes. It also depends on a distance function *d*(*x*, *y*) which measures the distance between the expression profiles *x* and *y* of two samples. The distance function utilises only the genes in the selected set of features *F*. To classify a sample with expression profile *y*, compute *d*(*x*, *y*) for each sample *x* in the training set. The predicted class of *y* is the class of the sample in the training set that is closest to *y* with regard to the distance function *d*. A variant of nearest neighbour classification is *k*-nearest neighbour classification. For example, with 3-nearest neighbour classification, you find the three samples in the training set that are closest to the sample *y*. The class that is most represented among these three samples is the predicted class for *y*. Nearest centroid, classification represents each class by a centroid, which is the average expression vector for all the arrays in that class. With nearest centroid classification, the test sample *y* is assigned to the class whose centroid it is closest to. The PAM method of Tibshirani *et al* (2002) is a variant of nearest centroid classification.

Dudoit *et al* also studied some more complex methods such as Classification Trees and aggregated classification trees. These methods did not appear to perform any better than diagonal linear discriminant analysis or nearest neighbour classification. Ben-Dor *et al* (2000) also compared several methods on several public data sets and found that nearest neighbour classification generally performed as well or better than more complex methods.

Complex methods with large numbers of parameters often fit the data used to develop the model well, but provide inaccurate predictions for independent data. This is often referred to as ‘overfitting’ the training data. Complex models have so many parameters that they can fit all of the random variations in the training data well. They find predictors and nonlinear functions that account for the random variations in the training data, but these discovered relationships do not represent real effects that exist in independent data; consequently, predictive ability is poor.

### Multiple-class prediction

**Figure 2:**
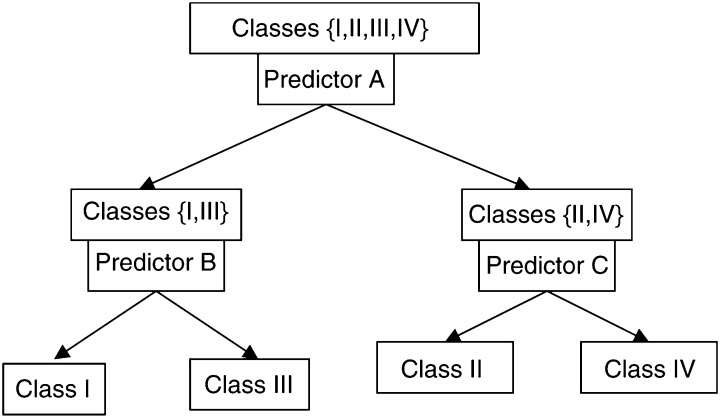
Tree structured classifier for predicting the unknown class of a tissue specimen when four classes are possible. Binary classifier A based on gene expression profile is first used to predict whether the specimen is in subset {I,III} or in subset {II,IV} of classes. For those specimens predicted to be in subset {I,III}, binary classifier B is used to predict whether the specimen is in class I or III. For those specimens predicted to be in subset {II,IV} based on classifier A, binary classifier C is used to predict whether the specimen is in class II or IV. The three binary classifiers will generally utilise different gene sets for prediction.

Many of the methods described above are for prediction when there are two classes. One strategy for multiple-class prediction is to perform a series of two-class predictions. The multiple-class prediction problem may be represented as a directed binary graph such as shown in Figure 2

. The root node at the top of the tree contains all of the classes and partitions them into two subsets based on a binary classifier. This type of binary separation is repeated for each subsequent node. Each node separates the set of classes input to it into two subsets based on a binary class predictor. The structure of the decision tree may be either determined based on pre-existing knowledge of the structure of the tissues being classified, or may be optimised computationally. There are several advantages to the use of tree structured binary classifiers. First, the wealth of methods for binary class prediction may be utilised. The binary decision problem at each node may be solved by any class prediction algorithm. Second, different feature (gene) sets may be used at different nodes. This approach does not require that one set of genes be utilised for the entire multi-class problem.

There are many alternative approaches to multi-class prediction. The *k*-nearest neighbour methods described above are not restricted to two classes. Since this method generally performs well and is one of the simplest, it is probably the most favoured of the non-tree-structured methods.

## DISCUSSION

DNA microarrays are a technology that offers great promise for refining diagnostic classification in ways that will enhance the efficiency of clinical trials and will enable the appropriate treatment to be matched to the appropriate patient. There are, however, major obstacles to the effective use of this technology. Access to tissue with viable RNA complicates research studies because the assay cannot be performed with conventional archived specimens. It also makes tissue acquisition and handling more difficult in the clinic. Another limitation is that most laboratories are not equipped to effectively design studies or analyse the immense amount of noisy data generated by DNA microarrays. The problems go far beyond data management, and include the difficulty organisations have in supporting interdisciplinary collaboration in a manner that brings the best minds together to educate each other and work creatively to make biological discoveries and solve important biomedical problems. The advent of DNA microarray technology has highlighted some of the problems in the area of data analysis. Potentially misleading analyses are published in the best biomedical journals and there is a plethora of algorithms and software of limited utility, sometimes questionable validity, and often based on inadequate understanding of the biological problems being addressed. These are growing pains, however, in a field that is dynamic, destined to make important biological discoveries and potentially revolutionise the process of developing cancer therapeutics.
